# Affective Enhancement of Working Memory Is Maintained in Depression

**DOI:** 10.1037/emo0000306

**Published:** 2017-04-13

**Authors:** Susanne Schweizer, Lauren Navrady, Lauren Breakwell, Rachel M. Howard, Ann-Marie Golden, Aliza Werner-Seidler, Tim Dalgleish

**Affiliations:** 1 Cognition and Brain Sciences Unit, Medical Research Council, Cambridge, United Kingdom; 2 Division of Psychiatry, University of Edinburgh; 3 Cognition and Brain Sciences Unit, Medical Research Council; 4 Cognition and Brain Sciences Unit, Medical Research Council; 5 The Black Dog Institute, Prince of Wales Hospital, Sydney, Australia, and Faculty of Medicine, University of New South Wales; 6 Cognition and Brain Sciences Unit, Medical Research Council, and Cambridgeshire and Peterborough NHS Foundation Trust, Cambridge, United Kingdom

**Keywords:** working memory capacity, emotion, depression, complex span

## Abstract

We currently know little about how performance on assessments of working memory capacity (WMC) that are designed to mirror the concurrent task demands of daily life are impacted by the presence of affective information, nor how those effects may be modulated by depression—a syndrome where sufferers report global difficulties with executive processing. Across 3 experiments, we investigated WMC for sets of neutral words in the context of processing either neutral or affective (depressogenic) sentences, which had to be judged on semantic accuracy (Experiments 1 and 2) or self-reference (Experiment 3). Overall, WMC was significantly better in the context of depressogenic compared with neutral sentences. However, there was no support for this effect being modulated by symptoms of depression (Experiment 1) or the presence of recurrent major depressive disorder (MDD; Experiments 2 and 3). Implications of these findings for cognitive theories of the role of WM in depression are discussed in the context of a growing body of research showing no support for a differential impact of depressogenic compared with neutral information on WM accuracy.

Individuals suffering from major depressive disorder (MDD) reliably show difficulties with executive functioning (Snyder, 2013). Yet the origin and impact of these difficulties on everyday mental life remains underexplored. Executive functioning problems, especially in affective disorders, have been proposed to arise as a result of cognitive resources being preferentially deployed toward the processing of affectively salient material (Gotlib & Joormann, 2010). That is, resources are recruited by affectively laden thoughts, feelings, and behavioral impulses, which although relevant to individuals’ immediate goal states (e.g., attempting to reduce negative affect), are often less relevant to the executive task at hand and therefore have the capacity to hinder performance on that task (Mason et al., 2007; Pessoa, 2009). Interestingly, however, until recently these executive functions have typically been investigated in the laboratory using affect-neutral tasks (Snyder, 2013). Explicitly investigating executive functioning within the context of affective information in the laboratory arguably provides a more naturalistic evaluation of the problems that individuals suffering from emotional disorders have with everyday mental operations in the context of their negative mood states and associated cognitions and behavioral tendencies (Schweizer & Dalgleish, 2011, 2016).

## Affective Working Memory in MDD

Preliminary advances in the investigation of executive functioning in affective contexts have been made in the area of working memory (WM), a capacity-limited system that consists of an executive control component interacting with one or more storage systems to transiently maintain and store information in the service of other forms of cognition (Baddeley, 2003). Specifically, WM performance in MDD has been assessed in contexts where either affective material has to be remembered as part of the task (e.g., Joormann & Gotlib, 2008) or where the affective material is present in the form of task-irrelevant distractor stimuli (e.g., Bertocci et al., 2012). These studies have produced equivocal findings. No studies found support for a differential impact of affective (positive or negative) material on WM *accuracy* in individuals currently suffering from MDD (Berman et al., 2011; Bertocci et al., 2012; Foland-Ross et al., 2013; Joormann & Gotlib, 2008; Joormann, Levens, & Gotlib, 2011; Joormann, Nee, Berman, Jonides, & Gotlib, 2010; Ladouceur et al., 2005; Levens & Gotlib, 2009, 2010; Tavitian et al., 2014; Yoon, LeMoult, & Joormann, 2014)^1^ nor in those in remission from their latest episode of MDD (De Lissnyder et al., 2012; Demeyer, De Lissnyder, Koster, & De Raedt, 2012; Kerestes et al., 2012; Levens & Gotlib, 2015) compared with healthy controls. For some of these tasks, WM *reaction time* (RT) appeared to be a more sensitive measure of WM performance with individuals suffering from MDD showing slowed RTs on WM tasks in the context of negative material compared with positive and/or neutral information (De Lissnyder et al., 2012; Demeyer et al., 2012; Joormann et al., 2011, 2010; Ladouceur et al., 2005; Levens & Gotlib, 2010, 2015; Yoon et al., 2014). However, even in the case of RT, the data was mixed with several studies finding no support for affective influences (Foland-Ross et al., 2013; Joormann & Gotlib, 2008; Levens & Gotlib, 2009; Tavitian et al., 2014).

## Is Affective Working Memory Capacity a More Sensitive Measure?

A potential account of these equivocal findings is that the WM measures employed (such as the *n*-back task and the modified Sternberg task) were measures of WM storage rather than assessments of what has been called WM capacity (WMC; Conway et al., 2005). WMC tasks seek to index the executive control component of WM described above, as opposed to simply storage. That is, individuals’ capacity to attend and respond to task-relevant information while inhibiting attention and prepotent responses to task-irrelevant distractions (Engle, 2002; Engle & Kane, 2003). WMC has been shown to be predictive of individual differences on a large number of higher-cognitive functions (Barrett, Tugade, & Engle, 2004). Individual differences in performance on a task assessing WMC in the context of affective distraction are therefore arguably predictive of functioning on a variety of cognitive operations (e.g., remembering the grocery list or keeping track of proceedings at a work meeting) requiring WMC in everyday life in affective contexts, such as depressed feeling states and thoughts.

Preliminary research in analogue samples examining the effects of depressed mood on WMC in the context of affective information appear to provide clearer support for the view that the presence of depressive symptoms would modulate the effects of affective material on WMC. In particular, a recent pair of studies showed that affective WMC in dysphoric undergraduates was impaired compared with nondysphoric students on an affective complex span task (Hubbard, Hutchison, Hambrick, & Rypma, 2016; Hubbard, Hutchison, Turner, et al., 2016). Complex span tasks are considered a gold-standard for operationalizing WMC and are defined as tasks that require simultaneous performance of a target storage task and some form of operation task (Conway et al., 2005). In both studies, Hubbard and colleagues showed that dysphoric participants remembered fewer numbers (storage task) than nondysphoric controls when they were required to simultaneously make judgments of self-relevance about depressogenic statements derived from symptom measures of depression (Beck, Ward, Mendelson, Mock, & Erbaugh, 1961; Lako et al., 2014; Radloff, 1977). There were no group differences in numbers recalled when the operation task required participants to make semantic decisions about neutral sentences. While these findings suggest that measures of WMC in affective contexts may be most sensitive to WM deficits reported in MDD, there are two methodological considerations that together merit a note of caution. First, it is unclear differential performance across the conditions was due to differences in the affective significance of the task stimuli (cf., Pessoa, 2009) or differences in the type of decision made (i.e., a semantic judgment vs. a self-relevance judgment). Second, these studies were conducted in seemingly high functioning healthy individuals (i.e., undergraduates) who reported dysphoric mood states. The underlying processes leading to WM deficits in these dysphoric students on this task may therefore be markedly different to the processes operating in MDD.

## The Present Study

In the present series of experiments, we therefore aimed to address the question of whether there is impact of affective information on WM in MDD when performing an affective WMC task (aWMC-task). We used the affective reading span task (Schweizer & Dalgleish, 2011), modified to include depressogenic material. The task required participants to recall words presented at the end of either neutral or depressogenic statements, which they had to judge in both cases in terms of semantic accuracy (Experiments 1 and 2) or self-relevance (Experiment 3). The task has previously been shown to be sensitive to differences in WMC in affective context between healthy individuals and those with emotional disorders (i.e., posttraumatic stress disorder (PTSD); Schweizer & Dalgleish, 2011). Specifically, in line with the dual competition framework (Pessoa, 2009) healthy individuals showed improved WMC for words following affective (relative to neutral) task-relevant information. The enhancement effect, however, was shown to be attenuated in individuals with a lifetime history of PTSD (Schweizer & Dalgleish, 2011).

Pessoa’s (2009) dual competition framework of the impact of affective significance on executive control can also be used to guide the predictions for the current experiments. According to the theory, low-threat (i.e., affective significance) affective information is prioritized at both the perceptual and executive levels of processing, thereby improving executive performance on tasks that include task-relevant affective information (Pessoa, 2009; Vuilleumier, 2005). The framework further suggests that stimuli high in affective significance hijack executive control resources thereby impairing performance on concurrent executive tasks. A recent meta-analysis has generated broad support for these predictions (Schweizer et al., 2016).

Based on the dual competition framework, we predicted that WMC in the presence of depressogenic compared with neutral sentences should be improved overall in a nonclinical community sample. However, we further hypothesized that individuals with high levels of depressive symptoms in that sample (Experiment 1) and those with MDD (Experiments 2 and 3) would be more impaired by negative, depressogenic sentences compared with those with lower levels of symptoms (Experiment 1) or never-depressed controls (Experiments 2 and 3), because the negative sentences arguably hold greater affective significance for depressed individuals.

## Experiment 1

Experiment 1 tested the hypothesis that performance on the aWMC-task would vary as a function of the valence (depressogenic vs. neutral) of the sentences that proceeded the memoranda. Based on the dual competition framework (Pessoa, 2009) reviewed above, we predicted that overall participants would show better WMC capacity in the presence of depressogenic material relative to neutral material (Hypothesis 1). However, aWMC (operationalized as performance in the depressogenic condition after subtracting performance in the neutral condition) was also predicted (Hypothesis 2) to show a negative association with depressive symptoms, as depressive symptomatology arguably increases the distractor sentences’ affective significance. That is, we expected those with higher levels of depressive symptoms to be more impaired in their WMC in the context of depressogenic sentences relative to neutral. We investigated these hypotheses in a community sample selected to provide a range of self-reported depression symptom severity across the non- to mild to severely depressed range. To further approximate a clinical case-control design, we also investigated whether individuals in this community sample scoring in the “depressed” range on the Beck Depression Inventory (BDI-II; Beck, Steer, & Brown, 1996) would perform differently on the aWMC-task compared with those scoring in the nondepressed range.

### Method

#### Participants

Exactly 123 participants (age range: 18–64 years, *M* = 41 years, *sd* = 14.21, 83 women) were recruited from the Medical Research Council Cognition and Brain Sciences Unit volunteer panel. To be included, participants needed to be between 18 and 65 years old, speak English with native fluency, have normal (or corrected to normal) vision, and be neurologically healthy (no history of neurological disorders or head injuries). Because of the focus of the study on depressive symptomatology, we used a stratified approach to recruit participants from across the whole range of scores on the BDI using scores recorded during previous assessments as an initial screen. The scale was then readministered in the current study. Specifically, we sourced participants classified as “nondepressed” (BDI scores of 0–13), alongside participants in the “depressed” range (>14), based on these previous BDI scores (Dozois, Dobson, & Ahnberg, 1998; Segal, Coolidge, Cahill, & O’Riley, 2008). The included sample presented with a good range of BDI scores (0–36; the maximum range is 0–63), with *n* = 83 (67.5%) in the nondepressed range, and *n* = 40 (32.5%) in the depressed range (of whom some, *n* = 3 (2.4%), would be classified as severe in clinical terms).

#### Measure: aWMC-task

To measure aWMC, we modified the valenced reading span task from Schweizer and Dalgleish (2011). In keeping with the original reading span task (Daneman & Carpenter, 1980), the valenced version is a complex span task (Conway et al., 2005), which requires the storage of single words (for a detailed description of the words included see: Schweizer & Dalgleish, 2011), in tandem with an operation component (evaluating sentences on their semantic accuracy) that potentially disrupts participants’ ability to memorize the to-be-remembered material. Valence is manipulated by including blocks of neutral and negative sentences. In this version of the aWMC-task, the negative sentences were relevant to depression and were derived from the 100-item Dysfunctional Attitude Scale (DAS; Weissman & Beck, 1978). The DAS has been shown to be sensitive to cognitive, genetic, and physiological vulnerabilities to depression (Beevers, Strong, Meyer, Pilkonis, & Miller, 2007; Meyer et al., 2003; Oliver, Murphy, Ferland, & Ross, 2007; Zuroff, Igreja, & Mongrain, 1990). Individuals with a history of depression endorse more items from the DAS but only if they are in a negative mood state (Miranda & Persons, 1988; Miranda, Persons, & Byers, 1990). Moreover, responses on the DAS before and after a negative mood induction are predictive of subsequent relapse in individuals remitted from depression (Segal et al., 2006). Statements from the DAS then should carry great affective significance for individuals experiencing depressed mood or suffering from MDD. The neutral sentences were created by the authors to match the depressogenic sentences in length and reading ease.

The task comprised between 4 and 7 (trial size) sentence-word pairings within each trial. Each trial size was presented twice for each valence. This resulted in 44 neutral and 44 depressogenic pairings, across eight trials for each valence. Participants were first presented with the sentences and had to indicate by saying “yes” or “no” whether the sentences were either semantically correct, or rendered nonsensical by the insertion of an irrelevant word into the sentence (e.g., “Sometimes it seems such an effort to do anything **parcel**”; bold font for illustration only). Each sentence was presented simultaneously with an unrelated upper case word of neutral valence displayed to the right of the sentence. These words had to be memorized for recall at the end of the trial. After rating the final sentence-word pairing in a given trial, participants were asked to recall the upper case neutral words presented after each sentence. They were instructed to recall the words in their presented order.

The proportions of words recalled correctly were computed for each trial size and valence and then collated to give proportions for depressogenic and neutral conditions across all trials, irrespective of whether they were recalled in the correct position or not, were computed for the neutral and depressogenic conditions separately. The aWMC index score was computed by subtracting the proportion of the words recalled in the neutral from the proportion recalled in the depressogenic condition (i.e., lower aWMC scores represent greater impairment by depressogenic distractors).

#### Measure: Symptoms of depression

Depressive symptomatology was assessed with the BDI-II (Beck et al., 1996), a well-validated 21-item inventory of affective, cognitive and physical symptoms in depression (Arnau, Meagher, Norris, & Bramson, 2001).

#### Measure: Verbal IQ

Error scores on the National Adult Reading Test (NART; Nelson, 1982) were used as a measure of verbal intelligence to allow us to evaluate whether any differences in WMC were due to differences in verbal intelligence.

#### Procedure

The following procedure was broadly consistent across all three experiments; any minor deviations are noted in the appropriate sections. After providing informed consent, participants completed the BDI-II and NART, before performing the aWMC-task. Participants were compensated for their time (£6 per hour). All 3 experiments were approved by the Cambridge Local Research Ethics Committee.

### Results and Discussion

Given research showing that WMC deteriorates with age (e.g., Campbell & Charness, 1990) and the possible influence of verbal IQ on this verbal version of the WMC^2^ task, we first explored potential correlations between the measures. aWMC correlated significantly with neither age, *r*(123) = −.14 nor verbal IQ *r*(112)^3^ = .03, with trivial effect sizes, and so we did not include either variable in our regression modeling.

Overall, in line with our first hypothesis, participants showed significantly better WMC for words following depressogenic (*M* = .54, *SD* = .18) compared with neutral (*M* = .50, *SD* = .16) sentences, *F*(1, 122) = 22.17, *p* < .001, η_p_^2^ = 0.15. The moderate to large effect was confirmed with Bayesian analysis. The posterior probability of an effect of valence was *p*(H1, *D*) = 1. The posterior probability of the experimental hypothesis indicates the likelihood of the effect being significant if this analysis were repeated in a different hypothetical sample. That is, the likelihood for an effect of valence on WMC in a different hypothetical sample is 100%. As noted in the Introduction, this is in line with Pessoa’s (2009) dual competition framework for the effects of affective significance on executive control performance. These findings further concur with evidence from the long-term memory literature, which shows a significant memory enhancement for affective stimuli and events (Buchanan & Adolphs, 2002; Phelps, 2004) as well as with our previous work in PTSD showing an affective enhancement effect on this task in trauma-exposed participants (Schweizer & Dalgleish, 2011). Accuracy on the semantic judgment task was high (98%), and did not differ across valence (neutral: *M* = .99, *SD* = .09; depressogenic: *M* = .98, *SD* = .04), *F*(1, 122) = 1.70, *p* = .20, η_p_^2^ = .01. This was confirmed by Bayesian analyses with the Bayes Factor providing moderate support for the null hypothesis, BF_01_ = 3.22. That is, the effects of valence on WMC accuracy are unlikely to be accounted for by the semantic decision being more cognitively demanding across valence conditions.

In contrast with our second hypothesis, symptoms of depression did not significantly predict aWMC,^4^ *b* = .001, 95% CI [−0.001, 0.002], *t* = 0.71, *p* = .478, *R*^2^ = .005. In order to examine the extent to which the null hypothesis of no effect of depressive symptoms on aWMC could be supported, this absence of an effect was again investigated using Bayesian analysis. The Bayes Factor for the null hypothesis was BF_01_ = 3.89 whereas the posterior probability an effect of depression was *p*(H1, *D*) = 0.20. The BF_01_ here provides moderate (values between 3 and 10 are considered to provide moderate support) evidence in support of the null hypothesis (Jeffreys, 1998). And the likelihood of finding a significant effect of depressive symptoms on aWMC in a different hypothetical sample was only 20%.

Finally, a comparison of aWMC in individuals scoring in the depressed range (above the cutoff of ≥14 on the BDI-II; Carney, Ulmer, Edinger, Krystal, & Knauss, 2009; Leentjens, Verhey, Luijckx, & Troost, 2000; *n* = 40, *M*_*BDI*_ = 20.63, *SD* = 5.49) with those who scored in the nondepressed range (*n* = 83; *M*_*BDI*_ = 5.28, *SD* = 4.51) revealed no significant effect of BDI-cutoff group on aWMC *F* ≤ 1, η_p_^2^ = 0.01 (see Table 1). These results were again supported by Bayesian analyses (see Table 1).[Table-anchor tbl1]

These findings were surprising because, based on the dual competition framework, we expected the affective significance of the negative statements to increase as a function of depressive symptomatology and aWMC to concurrently deteriorate. Moreover, we had expected the negative sentences to engender depressogenic (e.g., ruminative) processing in those with depressive tendencies and thereby recruit cognitive resources away from the storage component of the aWMC-task. The results are also at odds with the complex span data in dysphoric samples reported previously (Hubbard, Hutchison, Hambrick, et al., 2016; Hubbard, Hutchison, Turner, et al., 2016), although as noted there are also methodological issues surrounding the design of those studies. However, it should be reiterated that the current pattern of data is consistent with other studies looking at WM in affective contexts in depression using simple span WM tasks (Berman et al., 2011; Bertocci et al., 2012; De Lissnyder et al., 2012; Demeyer et al., 2012; Foland-Ross et al., 2013; Joormann & Gotlib, 2008; Joormann et al., 2011, 2010; Ladouceur et al., 2005; Levens & Gotlib, 2009, 2010, 2015; Tavitian et al., 2014; Yoon et al., 2014).

One possible account of the present finding is that these ruminative processes and other mechanisms, which may impact on WMC, only impair concurrent cognitive processing in those with recurrent clinical depression, which is associated with more ruminative responses compared with subclinical dysphoria (Garnefski et al., 2002). That is, the increased (relative to subclinical depression) tendency to ruminate on the depressogenic sentences reduces the cognitive resources available to perform the WM task. Specifically, rumination and WMC are both dependent on executive control processes (Cooney, Joormann, Eugène, Dennis, & Gotlib, 2010; Demeyer et al., 2012). We therefore explored aWMC-task performance in individuals with a current diagnosis of recurrent MDD compared with never-depressed individuals.

## Experiment 2

Experiment 2 employed a case-control design using the same procedure as Experiment 1 to compare aWMC in never-depressed participants against individuals with a diagnosis of recurrent MDD. We predicted greater aWMC in the never depressed individuals (ND group) relative to individuals in the MDD group.

### Method

#### Participants

Participants with recurrent MDD were recruited through newspaper and other local advertisements. Never-depressed participants were recruited through the departmental volunteer panel. Recurrent MDD was assessed upon arrival for testing using the Structured Clinical Interview for the *Diagnostic and Statistical Manual of Mental Disorders* (SCID; First, Spitzer, Gibbon, & Williams, 1995). The total sample in Experiment 2 included 36 participants (age range: 19–65, *M* = 51 years, *sd* = 10.77; 17 women), with 14 in the ND group and 22 in the MDD group. For one participant in the MDD group, the computer malfunctioned. The final sample therefore included 21 MDD participants (of whom 13 were experiencing a current episode of depression and 8 were in remission).

The groups did not significantly differ in age or verbal IQ (*F* < 1). However, compared with the ND group (93%, *n* = 13), there were significantly fewer women in the MDD group (50%, *n* = 11), Χ^*2*^ (1) = 5.19, *p* = .023 (with Yates continuity correction). Subsequent group comparisons therefore included gender as a covariate.

### Results and Discussion

In line with Experiment 1, there was a significant effect of valence (neutral: *M* = .46, *SD* = .16; depressogenic: *M* = .49, *SD* = .20) on WMC with better performance in the presence of depressogenic sentences, *F*(1, 34) = 4.88, *p* = .034, η_p_^2^ = 0.13, *p*(H1, *D*) = .63. As in Experiment 1, the effect of valence on WMC accuracy does not seem to be accounted for by differential difficulty of the semantic decision across conditions with high overall accuracy (97%), and no significant difference across conditions (neutral: *M* = .96, *SD* = .04; depressogenic: *M* = .97, *SD* = .04), *F*(1, 34) = 3.32, *p* = .08, η_p_^2^ = .09. Though it should be noted, Bayesian analyses supported neither experimental BF_10_ = 1.09 nor null hypothesis, BF_01_ = 0.99. There was no significant effect of group, *F*(1, 32) = 1.434, *p* = .240, η_p_^2^ = 0.04; *p*(H1, *D*) = .35 on WMC. Finally, failing to support our prediction, there was no significant interaction between group and valence,^5^ *F*(1, 32) < 1, η_p_^2^ = 0.02 with scores on the index of aWMC (WMC in depressogenic contexts minus WMC in neutral contexts) indicting comparable enhancement in depressogenic contexts across the MDD and ND groups (see Table 1 for means and Bayesian statistics). Because the MDD group also included individuals who were not currently experiencing an episode (*n* = 8), we repeated these analyses comparing the ND group only to individuals who were currently in episode. Again, there was no significant difference between groups on aWMC, *F*(1, 25) < 1; BF_01_ = 4.37; *p*(H1, *D*) = .19 (for means and effects of valence and group on WMC, see online supplemental materials). In other words, there was strong support for a null effect and the likelihood of an effect of diagnosis of MDD (current vs. never) on aWMC in a different sample was only 19%.

As noted, the absence of an effect of recurrent MDD on aWMC in Experiment 2 and the lack of association between aWMC and depressive symptoms in Experiment 1 is in contrast with Hubbard and colleagues (Hubbard, Hutchison, Hambrick, et al., 2016; Hubbard, Hutchison, Turner, et al., 2016), who showed a dissociation of WMC performance for affective compared with neutral contexts in their dysphoric student samples. A possible account for this discrepancy may be that the material in the present experiments was processed in a semantic rather than self-referential manner (Wisco, 2009). This hypothesis was investigated in Experiment 3.

## Experiment 3

Self-referential processing in depression has been argued to activate self-schemas and bias information processing toward self-referent material (Alloy, Abramson, Murray, Whitehouse, & Hogan, 1997). Previous research in individuals with depression has shown a recall advantage for words that were previously processed in a self-referential manner (Alloy et al., 1997; Kuiper & Derry, 1982; Kuiper, Olinger, MacDonald, & Shaw, 1985). In line with this, the long-term memory literature shows a mood-congruent recall bias for negative, depressogenic information in depression (for reviews see: Dalgleish & Watts, 1990; Gotlib & Krasnoperova, 1998), particularly for the most self-referent of memories: autobiographical memories (see Dalgleish & Werner-Seidler, 2014).

Here we investigated the effect of self-referential processing of depressogenic material on aWMC. We used the same case-control design as for Experiment 2 but modified the operation component of the aWMC-task. In this third experiment, participants were required to make judgments of self-reference instead of semantics regarding the statements that they read as part of the operation task. As the memoranda in the current task (the neutral words presented alongside the sentences) were unrelated to the sentences that required a self-reference judgment, the expectation was that drawing attention toward the sentences would therefore impair memory for the storage component of the aWMC.

In addition to these competing hypotheses regarding the effect of self-referential processing on aWMC in those with MDD currently in episode, in this study we also chose to explore aWMC in individuals with MDD in remission. It is well established that remitted depressed individuals present with patterns of endorsement for potentially self-referent depressogenic sentences on the DAS that are similar to those who have never been depressed (Eaves & Rush, 1984; Miranda, Gross, Persons, & Hahn, 1998; Miranda & Persons, 1988; Miranda et al., 1990; Segal et al., 2006; Segal, Shaw, Vella, & Katz, 1992).^6^ However, we also know that these nonendorsements of depressogenic statements are considerably slower to generate in remitted individuals relative to comparison conditions suggesting that nonendorsement of such statements in those in remission from depression comes with an executive control cost, arguably to do with inhibiting prepotent tendencies to endorse such sentences (Sheppard & Teasdale, 2004; Teasdale, 1988). We therefore sought to explore whether this putative cost would impact WMC in the context of self-referent processing of depressogenic sentences in remitted individuals, relative to neutral sentences and to the performance of the never-depressed comparison group.

### Method

#### Participants

Recruitment procedures were identical to Experiment 2 with the exception that we removed the upper age limit^7^ for Experiment 3, as there was no evidence that age was impacting the results. This resulted in 70 participants (age range: 18–76, *M* = 45, *SD* = 15, 48 women) comprising 20 never-depressed participants (ND group) and 50 participants with a diagnosis of recurrent MDD. Twenty-seven of the latter group were diagnosed with a current major depressive episode (MDD_C_-group), and 23 were currently in remission (MDD_R_-group) according to the SCID. The three groups did not significantly differ in verbal IQ or gender distribution. There was, however, a significant difference in age, *F*(1, 67) = 5.21, *p* = .008, *d* = 0.79 with the remitted group (*M* = 51.17, *SD* = 16.77) being the oldest and individuals in the MDD_C_-group (*M* = 44.15, *SD* = 14.30) being older than the ND group (*M* = 39.95, *SD* = 13.21). All group comparisons therefore included age as a covariate.

#### aWMC-task

As before, the aWMC-task required the storage of single words (presented at the end of each sentence), which had to be recalled at the end of each trial. In tandem with the storage task, participants had to perform an operation task that potentially disrupts their ability to memorize the to-be-remembered material. In the self-reference version of the aWMC, the operation task probed participants to rate how much they agreed with the statement that they had just read out-loud (instead of making a semantic judgment about these sentences as in Experiments 1 and 2). Agreement ratings were provided on a Likert scale ranging from 1 (*not at all*) to 7 (*very much*)*.* The depressogenic trials used the same sentences that were used in the aWMC for Experiments 1 and 2. The neutral sentences were modified to include neutral statements about which participants could make a judgment of self-reference (e.g., “I plan out what I am going to do during the day” or “I try to get at least seven hours sleep every night”). As before, the neutral sentences were matched for length and reading ease with the depressogenic sentences.

### Results and Discussion

#### Self-reference ratings

**Figure 1 fig1:**
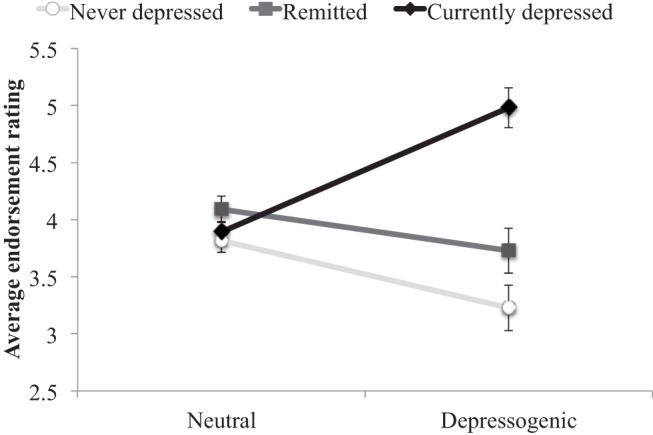
Ratings of endorsement of self-referent distractor statements. The figure shows participants’ average endorsement of the self-referent statements. Participants indicated on a scale from 1 (*not at all*) to 7 (*very much*) how much they agreed with the sentence about themselves.

As would be expected, analyses of the rating scores showed a significant interaction between group and endorsement, *F*(2, 66) = 30.92, *p* < .001, η_p_^2^ = 0.48, with the ND and MDD_R_-groups showing similar response patterns, and only the MDD_C_-group showing greater relative endorsement of depressogenic sentences (see Figure 1). The finding suggests that participants were processing the sentence content appropriately and that the sentences included were sensitive to active depressive concerns. Moreover, the finding is in line with research showing that endorsement of the DAS is mood-dependent (Miranda et al., 1990).[Fig-anchor fig1]

#### aWMC performance

In line with Experiments 1 and 2, when performance on endorsed and nonendorsed sentences were included together, there was a significant effect of valence (neutral: *M* = .52, *SD* = .16; depressogenic: *M* = .54, *SD* = .16) on WMC performance, *F*(1, 67) = 4.09, *p* = .047, η_p_^2^ = 0.06, with better performance again in the presence of depressogenic sentences. As in Experiment 2, there was no significant effect of group on WMC, *F*(2, 64) = 1.31, *p* = .244, η_p_^2^ = 0.01. The finding was confirmed by Bayesian analyses, BF_01_ = 2.19, *p*(H1, *D*) = .31.There was also no significant group by valence interaction, *F*(2, 64) = 1.21, *p* = .305, η_p_^2^ = 0.03, BF_01_ = 4.08, *p*(H1, *D*) = .20. Examining the aWMC scores again revealed comparable performance across the ND and the MDD groups with the absence of a group by valence interaction again confirmed by Bayesian analyses with moderate support for the null hypothesis BF_01_ = 4.08 and an interaction only likely to be found in 20% of hypothetical new samples, *p*(H1, *D*) = .20. See Table 1 for a comparison between the MDD groups combined and the ND group analogue to Experiment 2.

The group comparisons including only individuals currently suffering from MDD and the ND group showed a small effect of group (MDD_C_ vs. ND) on WMC, *F*(1, 41) = 1.57, *p* = .022, η_p_^2^ = 0.04. Bayesian analyses provided further support for an effect of group on WMC by rejecting the null hypothesis BF_01_ = 0.69 and yielding a 60% likelihood of finding and effect of group in a different hypothetical sample, *p*(H1, *D*) = .60. As in Experiment 2 there was, however, no significant group by valence interaction, *F*(1, 41) < 1, η_p_^2^ = 0.00, BF_01_ = 3.96, *p*(H1, *D*) = .20. The significant effect of group on WMC may be due to the self-reference task engendering a cycle of self-referential thinking in the MDD group but not the ND group. Moreover, it should be noted that, while it was not significant, the effect of group was moderate in size in Experiment 2, and in Experiment 1, there was a statistical trend for an association between symptoms of depression and overall WMC, *r*(123) = .17, *p* = .07. These equivocal findings should be considered in the broader literature on executive functioning in depression, which has revealed relatively robust deficits in depression (for a review see: Snyder, 2013). Additional insight into executive control alteration in depression and dysphoria will be gained from the growing neuroimaging literature (De Raedt, Koster, & Joormann, 2010; Owens, Koster, & Derakshan, 2012), which shows group differences even in the absence of behavioral differences (Kerestes et al., 2012).

Next, we examined whether there were differential effects for endorsed versus unendorsed sentences. That is, we compared proportion of words recalled correctly that followed endorsed neutral, nonendorsed neutral, endorsed depressogenic, and nonendorsed depressogenic statements (see Table 2). We found no support for a three-way interaction of Group × Valence × Endorsement,^8^ *F*(2, 64) <1; η_p_^2^ = 0.01; BF_01_ = 5.95; *p*(H1, *D*) = .14. Again the absence of an interaction was supported by the Bayesian analyses. Moreover, comparing only the MDD_C_ to the ND group yielded the same pattern of results, *F*(1, 41) <1; η_p_^2^ = 0.00; BF_01_ = 3.73; *p*(H1, *D*) = .21.[Table-anchor tbl2]

A potential account for the absence of a three-way interaction is that the affective salience of endorsed items is insufficient to adversely impact aWMC differentially in those with depression. Although the depressed group endorsed more of the depressogenic sentences as self-relevant, the mean strengths of endorsement were only *M* = 5.00^9^ (*SD* = 0.87) on a 7-point scale. Although lack of affective impact is therefore an important factor to consider, it should also be noted that, in the context of the differential activation hypothesis, discussed above, the impact of depressogenic statements on cognitive processing has been observed in other cognitive tasks in the absence of endorsement of these statements. For example, Sheppard and Teasdale (2004) showed that response latencies on a semantic decision making task in currently depressed and remitted individuals were comparable despite remitted individuals endorsing the statements less strongly compared with currently depressed individuals.

Finally, given the findings by Hubbard and colleagues (Hubbard, Hutchison, Hambrick, et al., 2016, Hubbard, Hutchison, Turner, et al., 2016), which showed a difference between groups only for the depressogenic condition, we investigated the interaction of the effects of group and endorsement in depressogenic trials only. Again there was no significant interaction between group and endorsement when including all three groups, *F*(2, 64) <1; η_p_^2^ = 0.02; BF_01_ = 5.06; *p*(H1, *D*) = .17, nor when comparing the MDD_C_ and ND groups only, *F*(1, 41) <1; η_p_^2^ = 0.02; BF_01_ = 2.18; *p*(H1, *D*) = .31.

### Meta-Analytic Investigation of the Effect Size Across Experiments 1–3

#### Valence

There was good support of an overall effect of valence across the three experiments with a pooled effect size of η_p_^2^ = 0.11, which translates to a Cohen’s *d* of 0.70. Bayesian analyses confirmed the conclusion from the pooled analyses with conclusive support for a rejection of the null hypothesis BF_01_ = 2.12^−5^ and a posterior probability of *p*(H1, *D*) = 1 for an effect of valence. That is, using this task an effect of valence on WMC should be found in 100% of hypothetical future samples.

#### Effect of clinical levels of depression on aWMC

aWMC was unaffected by clinical levels of depression as was evidenced by the pooled effect size across all three experiments of η_*p*_^2^ = 0.00 (Cohen’s *d* = 0.06). Again these meta-analytic results were confirmed by Bayesian analyses on the pooled sample with moderate support in favor of accepting the null hypothesis BF_01_ = 6.38 and a posterior probability of *p*(H1, *D*) = .14 for an effect of clinical depression on aWMC.

## General Discussion

The first aim of the present series of experiments was to investigate the hypothesis that WMC would be enhanced in the context of affective compared with neutral information. Second, we aimed to resolve the discrepant findings in the literature on the effects of depression on aWMC using a complex span task. In line with the first hypothesis, the results showed a moderate to large (η_p_^2^ = 0.06–0.15; i.e., Cohen’s *d* = 0.50 – 0.84) WM-recall advantage for words following depressogenic sentences (relative to neutral) across three different studies in analyses combining individuals with varying levels of depression (a meta-analytic review across the three experiments yielded a moderate effect size: Cohen’s *d* = .70). This was the case irrespective of whether the statements were judged on affect-neutral semantic characteristics (Experiments 1 and 2) or on their affectively laden level of self-reference (Experiment 3).The finding is in line with the dual competition framework arguing that across both perceptual and executive competition affective material will be preferentially processed thereby enhancing executive performance on tasks with task-relevant affective stimuli (Pessoa, 2009).

In contrast with the framework’s predictions, however, affective significance of the stimulus material did not appear to impact on WMC performance. That is, individuals with MDD for whom the DAS statements used in the depressogenic condition should have significantly greater affective significance did not differ in aWMC performance compared with never-depressed groups. The finding was reliable (i.e., pooled trivial effect size of Cohen’s *d* = 0.06 and BF_01_ = 6.38 in support of the null hypothesis) across all three studies again irrespective of whether the operation task required a semantic or self-reference judgment. The finding contributes to a growing body of literature providing little support for the differential effects of affective material on WM accuracy in depression (Berman et al., 2011; Bertocci et al., 2012; Foland-Ross et al., 2013; Joormann & Gotlib, 2008; Joormann et al., 2011, 2010; Ladouceur et al., 2005; Levens & Gotlib, 2009, 2010; Tavitian et al., 2014; Yoon et al., 2014).

Findings from Experiment 3 are, however, in contrast with Hubbard and colleagues (Hubbard, Hutchison, Hambrick, et al., 2016; Hubbard, Hutchison, Turner, et al., 2016) who showed an impairing effect of depressogenic statements (relative to neutral) on WMC in dysphoric undergraduates. While the present study offers support against an impairing effect, or attenuation of the enhancement effect in currently depressed individuals and those in remission from depression, it does not preclude such an impairing effect in subclinical dysphoria.

Interestingly, however, as noted in the Introduction, theories of WM in depression have been advanced on the assumption of such a WM deficit that is likely to be particularly pronounced for depressogenic material (Gotlib & Joormann, 2010; Koster, De Lissnyder, Derakshan, & De Raedt, 2011). One possibility is that, if such an effect exists, it may be more discernible when looking at performance on a complex span task that requires the inhibition of attention and responses toward truly task-irrelevant distractors as opposed to sentences that have to be processed as part of the task, as in the current design. Preliminary evidence to support this hypothesis stems from Schweizer and Dalgleish (2016) who showed that simultaneously performing a visuospatial search task (operation task) and a verbal storage task (remembering words) in the context of either neutral or negative task-irrelevant background images impaired WMC in both healthy and clinical (i.e., lifetime history of PTSD) samples reliably. In line with Schweizer and Dalgleish (2016), a recent meta-analysis investigating the effects of affective material on WM showed that negative distractors, in particular, had an impairing effect on WM performance (Schweizer et al., 2016).

Another possibility is that any effects of affective material on WM in depression may take the form of impoverished processing in the context of positive material, rather than differential processing of negative material (Rottenberg, 2007). However, to date there is little support for this view, with studies failing to show a differential effect of positive information on WM accuracy in those with depression compared with healthy controls (Bertocci et al., 2012; Joormann & Gotlib, 2008; Joormann et al., 2011; Kerestes et al., 2012; Ladouceur et al., 2005; Levens & Gotlib, 2009, 2010; Tavitian et al., 2014).

A further possibility is that, while WM accuracy may not be a sensitive measure of WMC deficits experienced in MDD, RT could be. Again as discussed at the outset, findings are equivocal with some (e.g., De Lissnyder et al., 2012) but not all (e.g., Foland-Ross et al., 2013) studies showing slowed WM RT for negative relative to neutral and/or positive material. One question that remains unanswered with this line of research into WM RT is how this relates to the executive functioning deficits experienced in everyday life. That is, a measure of WM accuracy is intuitively related to a quotidian task such as remembering a grocery list. In contrast, it is more difficult to hypothesize how differential RTs for negative versus neutral items on a WM task are related to remembering to buy dishwater tablets and milk.

The present study showed an enhancing effect of affective information on WMC in a pooled sample of nearly 300 participants, including over 100 participants endorsing clinically significant levels of depression, across three different experiments. Together the findings supported the hypothesis based on the dual competition framework (Pessoa, 2009) and insights from the long-term memory literature that *task-relevant* affective content improves WMC. However, in contrast with predictions from both the dual competition framework and theoretical models of cognitive vulnerabilities to depression, there was no support for reduced aWMC in those currently suffering from MDD, in remission from recurrent MDD, or who self-reported symptoms of depression above the clinical cutoff for depression.

## Supplementary Material

10.1037/emo0000306.supp

## Figures and Tables

**Table 1 tbl1:** WM Performance across Depressive State and Condition

	Experiment 1		Experiment 2		Experiment 3		
	<BDI-II cutoff *n* = 83	>BDI-II cutoff *n* = 40	Never depressed *n* = 14	MDD *n* = 13	Never depressed *n* = 19	Current MDD *n* = 25	Remitted MDD *n* = 24
Neutral *M* (*SD*)	.53 (.17)	.48 (.13)	.50 (.15)	.43 (.17)	.56 (.15)	.48 (.16)	.53 (.16)
Depressogenic *M* (*SD*)	.56 (.19)	.52 (.16)	.53 (.22)	.47 (.19)	.60 (.13)	.51 (.17)	.53 (.19)
aWMC	.03 (.09)	.04 (.08)	.03 (.12)	.04 (.09)	.04 (.12)	.04 (.09)	.00 (.10)
Effect sizes (η_p_^2^)							
Valence	η_p_^2^ = .15		η_p_^2^ = .13		η_p_^2^ = .06		
Group	η_p_^2^ = .02		η_p_^2^ = .04		η_p_^2^ = .01		
Valence × Group^a^	η_p_^2^ = .01		η_p_^2^ = .02		η_p_^2^ = .01		
	Bayes factor in support of null hypothesis (BF_01_) and posterior probability for the experimental hypothesis *p*(H1, *D*)						
Valence	BF_01_ = 4.53^−4^; *p*(H1, *D*) = 1		BF_01_ = .58; *p*(H1, *D*) = .63		BF_01_ = .88; *p*(H1, *D*) = .52		
WMC: Group	BF_01_ = 1.87; *p*(H1, *D*) = .35		BF_01_ = 1.87; *p*(H1, *D*) = .35		BF_01_ = 1.13; *p*(H1, *D*) = .47		
Valence × Group	BF_01_ = 3.25; *p*(H1, *D*) = .23		BF_01_ = 2.61; *p*(H1, *D*) = .28		BF_01_ = 3.26; *p*(H1, *D*) = .23		
*Note*. Neutral = proportion of words recalled correctly in the context of neutral sentences; depressogenic = proportion of words recalled correctly in the context of depressogenic sentences; < BDI-II cutoff = individuals scoring 14 or lower on the BDI-II; > BDI-II cutoff = individuals scoring 15 or higher on the BDI-II; never depressed = individuals with no history of MDD; current MDD = individuals currently suffering from MDD; remitted MDD = individuals in remission from MDD; valence = effect comparing negative versus neutral trials; group = effect of group on WMC; Valence × Group = interaction between valence and group.							
^a^ Effect size comparing never depressed vs. combined remitted and MDD for comparison with Experiment 1 and 2. For effect sizes across all three groups, please see main text.							

**Table 2 tbl2:** Proportion of Words Recalled across Valence, Group and Endorsement

Sentence type	Never depressed	Remitted MDD	Current MDD
Neutral endorsed *M* (*SD*)	.61 (.16)	.55 (.20)	.51 (.17)
Neutral not-endorsed *M* (*SD*)	.51 (.15)	.50 (.19)	.45 (.17)
Depressogenic endorsed *M* (*SD*)	.60 (.19)	.52 (.22)	.51 (.18)
Depressogenic not-endorsed *M* (*SD*)	.60 (.16)	.55 (.16)	.52 (.21)
*Note*. Neutral endorsed = proportion of correctly recalled words that followed neutral sentences, which participants endorsed (i.e., rated 5 or higher); neutral not-endorsed = proportion of correctly recalled words that followed neutral sentences, which participants did not endorse (i.e., rated 4 or less); depressogenic endorsed = proportion of correctly recalled words that followed depressogenic sentences, which participants endorsed (i.e., rated 5 or higher); depressogenic not-endorsed = proportion of correctly recalled words that followed depressogenic sentences, which participants did not endorse (i.e., rated 4 or less).			

## Footnotes

1 A study in a sample of youth with MDD showed impaired (relative to healthy controls) WM for neutral trials (Tavitian et al., 2014).

2 The correlations were also nonsignificant between BDI-II scores and the individual condition WMC scores (i.e., depressogenic WMC and neutral WMC), *r*’s (112) = −.05–.18, *p* values > .05.

3 NART scores were missing for 9 participants. However, given the nonsignificant association between verbal IQ and aWMC, which meant there was no case for covarying for verbal IQ, we included these participants in the overall analyses. We ran all analyses excluding these participants as well, and the pattern of results remained unchanged.

4 Differential accuracy (depressogenic—neutral) on the semantic operation task was not predicted by symptoms of depression, *b* = .000, 95% CI [−0.002, 0.002], *t* = 0.14, *p* = .89, *R*^2^ = .000.

5 Moreover, there was no significant group-by-valence interaction for accuracy in semantic decision making, *F*(1, 33) = 0.30, *p* = .59; η_p_^2^ = 0.01.

6 However see: Haeffel et al. (2005) for a failure to replicate the predictive validity of the DAS in a remitted sample.

7 We also ran all analyses excluding the 7 participants that were older than 65 years, and this did not change the pattern of results.

8 Percentage of sentences endorsed (i.e., rated 5 or higher) per valence: Depressogenic = .43, *SD* = .17; Neutral = .43, *SD* = .11.

9 It should be noted though that an endorsement of 5 is at the higher end of the spectrum for clinical groups, with currently depressed individuals’ typical endorsement score for DAS items ranging between 3.5–4.5 (e.g., Ohrt & Thorell, 1998; Rogers et al., 2009; Wang, Halvorsen, Eisemann, & Waterloo, 2010), though we appreciate that the endorsements within the context of a memory task cannot be directly compared with those using a pen and paper measure.

## References

[c1] Alloy L. B., Abramson L. Y., Murray L. A., Whitehouse W. G., & Hogan M. E. (1997). Self-referent information-processing in individuals at high and low cognitive risk for depression. Cognition and Emotion, 11, 539–568. 10.1080/026999397379854a

[c2] Arnau R. C., Meagher M. W., Norris M. P., & Bramson R. (2001). Psychometric evaluation of the Beck Depression Inventory-II with primary care medical patients. Health Psychology, 20, 112–119. 10.1037/0278-6133.20.2.112 11315728

[c3] Baddeley A. (2003). Working memory: Looking back and looking forward. Nature Reviews Neuroscience, 4, 829–839. 10.1038/nrn1201 14523382

[c4] Barrett L. F., Tugade M. M., & Engle R. W. (2004). Individual differences in working memory capacity and dual-process theories of the mind. Psychological Bulletin, 130, 553–573. 10.1037/0033-2909.130.4.553 15250813 PMC1351135

[c5] Beck A. T., Steer R. A., & Brown G. K. (1996). Manual for the Beck Depression Inventory-II. San Antonio, TX: Psychological Cooperation.

[c6] Beck A. T., Ward C. H., Mendelson M., Mock J., & Erbaugh J. (1961). An inventory for measuring depression. Archives of General Psychiatry, 4, 561–571. 10.1001/archpsyc.1961.01710120031004 13688369

[c7] Beevers C. G., Strong D. R., Meyer B., Pilkonis P. A., & Miller I. R. (2007). Efficiently assessing negative cognition in depression: An item response theory analysis of the Dysfunctional Attitude Scale. Psychological Assessment, 19, 199–209. 10.1037/1040-3590.19.2.199 17563201

[c8] Berman M. G., Nee D. E., Casement M., Kim H. S., Deldin P., Kross E., . . . Jonides J. (2011). Neural and behavioral effects of interference resolution in depression and rumination. Cognitive, Affective & Behavioral Neuroscience, 11, 85–96. 10.3758/s13415-010-0014-x PMC4006074 21264648

[c9] Bertocci M. A., Bebko G. M., Mullin B. C., Langenecker S. A., Ladouceur C. D., Almeida J. R. C., & Phillips M. L. (2012). Abnormal anterior cingulate cortical activity during emotional n-back task performance distinguishes bipolar from unipolar depressed females. Psychological Medicine, 42, 1417–1428. 10.1017/S003329171100242X 22099606 PMC3601380

[c10] Buchanan T. W., & Adolphs R. (2002). The role of the human amygdala in emotional modulation of long-term declarative memory. Advances in Consciousness Research, 44, 9–34. 10.1075/aicr.44.02buc

[c11] Campbell J. I., & Charness N. (1990). Age-related declines in working-memory skills: Evidence from a complex calculation task. Developmental Psychology, 26, 879–888. 10.1037/0012-1649.26.6.879

[c12] Carney C. E., Ulmer C., Edinger J. D., Krystal A. D., & Knauss F. (2009). Assessing depression symptoms in those with insomnia: An examination of the beck depression inventory 2nd ed. (BDI-II). Journal of Psychiatric Research, 43, 576–582. 18954876 10.1016/j.jpsychires.2008.09.002 PMC2677199

[c13] Conway A. R. A., Kane M. J., Bunting M. F., Hambrick D. Z., Wilhelm O., & Engle R. W. (2005). Working memory span tasks: A methodological review and user’s guide. Psychonomic Bulletin & Review, 12, 769–786. 10.3758/BF03196772 16523997

[c14] Cooney R. E., Joormann J., Eugène F., Dennis E. L., & Gotlib I. H. (2010). Neural correlates of rumination in depression. Cognitive, Affective & Behavioral Neuroscience, 10, 470–478. 10.3758/CABN.10.4.470 PMC4476645 21098808

[c15] Dalgleish T., & Watts F. N. (1990). Biases of attention and memory in disorders of anxiety and depression. Clinical Psychology Review, 10, 589–604. 10.1016/0272-7358(90)90098-U

[c16] Dalgleish T., & Werner-Seidler A. (2014). Disruptions in autobiographical memory processing in depression and the emergence of memory therapeutics. Trends in Cognitive Sciences, 18, 596–604. 10.1016/j.tics.2014.06.010 25060510

[c17] Daneman M., & Carpenter P. A. (1980). Individual differences in working memory and reading. Journal of Verbal Learning & Verbal Behavior, 19, 450–466. 10.1016/S0022-5371(80)90312-6

[c18] De Lissnyder E., Koster E. H. W., Everaert J., Schacht R., Van den Abeele D., & De Raedt R. (2012). Internal cognitive control in clinical depression: General but no emotion-specific impairments. Psychiatry Research, 199, 124–130. 10.1016/j.psychres.2012.04.019 22578821

[c19] Demeyer I., De Lissnyder E., Koster E. H. W., & De Raedt R. (2012). Rumination mediates the relationship between impaired cognitive control for emotional information and depressive symptoms: A prospective study in remitted depressed adults. Behaviour Research and Therapy, 50, 292–297. 10.1016/j.brat.2012.02.012 22449892

[c20] De Raedt R., Koster E. H., & Joormann J. (2010). Attentional control in depression: A translational affective neuroscience approach. Cognitive, Affective & Behavioral Neuroscience, 10, 1–7. 10.3758/CABN.10.1.1 20233951

[c21] Dozois D. J. A., Dobson K. S., & Ahnberg J. L. (1998). A psychometric evaluation of the Beck Depression Inventory–II. Psychological Assessment, 10, 83–89. 10.1037/1040-3590.10.2.83

[c22] Eaves G., & Rush A. J. (1984). Cognitive patterns in symptomatic and remitted unipolar major depression. Journal of Abnormal Psychology, 93, 31–40. 10.1037/0021-843X.93.1.31 6699272

[c23] Engle R. W. (2002). Working memory capacity as executive attention. Current Directions in Psychological Science, 11, 19–23. 10.1111/1467-8721.00160

[c24] Engle R. W., & Kane M. J. (2003). Executive attention, working memory capacity, and a two-factor theory of cognitive control In Ross B. (Ed.), Psychology of learning and motivation (Vol. 44, pp. 145–199). New York, NY: Academic Press 10.1016/S0079-7421(03)44005-X

[c25] First M. B., Spitzer R. L., Gibbon M., & Williams J. B. W. (1995). Structured Clinical Interview for DSM–IV Axis I Disorders-Patient Edition. New York, NY: Biometrics Research Department, New York State Psychiatric Institute.

[c26] Foland-Ross L. C., Hamilton J. P., Joormann J., Berman M. G., Jonides J., & Gotlib I. H. (2013). The neural basis of difficulties disengaging from negative irrelevant material in major depression. Psychological Science, 24, 334–344. 10.1177/0956797612457380 23334445 PMC4004633

[c27] Garnefski N., Van Den Kommer T., Kraaij V., Teerds J., Legerstee J., & Onstein E. (2002). The relationship between cognitive emotion regulation strategies and emotional problems: Comparison between a clinical and a non-clinical sample. European Journal of Personality, 16, 403–420. 10.1002/per.458

[c28] Gotlib I. H., & Joormann J. (2010). Cognition and depression: Current status and future directions. Annual Review of Clinical Psychology, 6, 285–312. 10.1146/annurev.clinpsy.121208.131305 PMC2845726 20192795

[c29] Gotlib I. H., & Krasnoperova E. (1998). Biased information processing as a vulnerability factor for depression. Behavior Therapy, 29, 603–617. 10.1016/S0005-7894(98)80020-8

[c30] Haeffel G. J., Abramson L. Y., Voelz Z. R., Metalsky G. I., Halberstadt L., Dykman B. M., . . . Alloy L. B. (2005). Negative cognitive styles, dysfunctional attitudes, and the remitted depression paradigm: A search for the elusive cognitive vulnerability to depression factor among remitted depressives. Emotion, 5, 343–348. 10.1037/1528-3542.5.3.343 16187869

[c31] Hubbard N. A., Hutchison J. L., Hambrick D. Z., & Rypma B. (2016). The enduring effects of depressive thoughts on working memory. Journal of Affective Disorders, 190, 208–213. 10.1016/j.jad.2015.06.056 26519641

[c32] Hubbard N. A., Hutchison J. L., Turner M., Montroy J., Bowles R. P., & Rypma B. (2016). Depressive thoughts limit working memory capacity in dysphoria. Cognition and Emotion, 30, 193–209. 10.1080/02699931.2014.991694 25562416

[c33] Jeffreys H. (1998). The theory of probability (3rd ed.). New York, NY: Oxford University Press.

[c34] Joormann J., & Gotlib I. H. (2008). Updating the contents of working memory in depression: Interference from irrelevant negative material. Journal of Abnormal Psychology, 117, 182–192. 10.1037/0021-843X.117.1.182 18266496

[c35] Joormann J., Levens S. M., & Gotlib I. H. (2011). Sticky thoughts: Depression and rumination are associated with difficulties manipulating emotional material in working memory. Psychological Science, 22, 979–983. 10.1177/0956797611415539 21742932 PMC11862919

[c36] Joormann J., Nee D. E., Berman M. G., Jonides J., & Gotlib I. H. (2010). Interference resolution in major depression. Cognitive, Affective & Behavioral Neuroscience, 10, 21–33. 10.3758/CABN.10.1.21 PMC2845922 20233953

[c37] Kerestes R., Ladouceur C. D., Meda S., Nathan P. J., Blumberg H. P., Maloney K., . . . Phillips M. L. (2012). Abnormal prefrontal activity subserving attentional control of emotion in remitted depressed patients during a working memory task with emotional distracters. Psychological Medicine, 42, 29–40. 10.1017/S0033291711001097 21733287

[c38] Koster E. H. W., De Lissnyder E., Derakshan N., & De Raedt R. (2011). Understanding depressive rumination from a cognitive science perspective: The impaired disengagement hypothesis. Clinical Psychology Review, 31, 138–145. 10.1016/j.cpr.2010.08.005 20817334

[c39] Kuiper N. A., & Derry P. A. (1982). Depressed and nondepressed content self-reference in mild depressives. Journal of Personality, 50, 67–80. 10.1111/j.1467-6494.1982.tb00746.x 7086630

[c40] Kuiper N. A., Olinger L. J., MacDonald M. R., & Shaw B. F. (1985). Self-schema processing of depressed and nondepressed content: The effects of vulnerability to depression. Social Cognition, 3, 77–93. 10.1521/soco.1985.3.1.77

[c41] Ladouceur C. D., Dahl R. E., Williamson D. E., Birmaher B., Ryan N. D., & Casey B. J. (2005). Altered emotional processing in pediatric anxiety, depression, and comorbid anxiety-depression. Journal of Abnormal Child Psychology, 33, 165–177. 10.1007/s10802-005-1825-z 15839495

[c42] Lako I. M., Wigman J. T., Klaassen R. M., Slooff C. J., Taxis K., & Bartels-Velthuis A. A. (2014). Psychometric properties of the self-report version of the Quick Inventory of Depressive Symptoms (QIDS-SR_16_) questionnaire in patients with schizophrenia. BMC Psychiatry, 14, 247 10.1186/s12888-014-0247-2 25178310 PMC4159524

[c43] Leentjens A. F. G., Verhey F. R. J., Luijckx G.-J., & Troost J. (2000). The validity of the Beck Depression Inventory as a screening and diagnostic instrument for depression in patients with Parkinson’s disease. Movement Disorders, 15, 1221–1224. 10.1002/1531-8257(200011)15:6<1221::AID-MDS1024>3.0.CO;2-H 11104209

[c44] Levens S. M., & Gotlib I. H. (2009). Impaired selection of relevant positive information in depression. Depression and Anxiety, 26, 403–410. 10.1002/da.20565 19347861 PMC2836936

[c45] Levens S. M., & Gotlib I. H. (2010). Updating positive and negative stimuli in working memory in depression. Journal of Experimental Psychology: General, 139, 654–664. 10.1037/a0020283 21038984 PMC2984552

[c46] Levens S. M., & Gotlib I. H. (2015). Updating emotional content in recovered depressed individuals: Evaluating deficits in emotion processing following a depressive episode. Journal of Behavior Therapy and Experimental Psychiatry, 48, 156–163. 10.1016/j.jbtep.2015.03.009 25889375 PMC4524779

[c47] Mason M. F., Norton M. I., Van Horn J. D., Wegner D. M., Grafton S. T., & Macrae C. N. (2007). Wandering minds: The default network and stimulus-independent thought. Science, 315, 393–395. 10.1126/science.1131295 17234951 PMC1821121

[c48] Meyer J. H., McMain S., Kennedy S. H., Korman L., Brown G. M., DaSilva J. N., . . . Links P. (2003). Dysfunctional attitudes and 5-HT2 receptors during depression and self-harm. The American Journal of Psychiatry, 160, 90–99. 10.1176/appi.ajp.160.1.90 12505806

[c49] Miranda J., Gross J. J., Persons J. B., & Hahn J. (1998). Mood matters: Negative mood induction activates dysfunctional attitudes in women vulnerable to depression. Cognitive Therapy and Research, 22, 363–376. 10.1023/A:1018709212986

[c50] Miranda J., & Persons J. B. (1988). Dysfunctional attitudes are mood-state dependent. Journal of Abnormal Psychology, 97, 76–79. 10.1037/0021-843X.97.1.76 3351115

[c51] Miranda J., Persons J. B., & Byers C. N. (1990). Endorsement of dysfunctional beliefs depends on current mood state. Journal of Abnormal Psychology, 99, 237–241. 10.1037/0021-843X.99.3.237 2212273

[c52] Nelson H. E. (1982). National Adult Reading Test (NART): Test manual. Windsor, UK: NFER-Nelson.

[c73] Ohrt T., & Thorell L. H. (1998). Dysfunctional Attitude Scale (DAS). Psychometrics and norms of the Swedish version. Scandinavian Journal of Behaviour Therapy, 27, 105–113. 10.1080/02845719808408501

[c53] Oliver J. M., Murphy S. L., Ferland D. R., & Ross M. J. (2007). Contributions of the cognitive style questionnaire and the dysfunctional attitude scale to measuring cognitive vulnerability to depression. Cognitive Therapy and Research, 31, 51–69. 10.1007/s10608-006-9067-0

[c54] Owens M., Koster E. H. W., & Derakshan N. (2012). Impaired filtering of irrelevant information in dysphoria: An ERP study. Social Cognitive and Affective Neuroscience, 7, 752–763. 10.1093/scan/nsr050 21896495 PMC3475351

[c55] Pessoa L. (2009). How do emotion and motivation direct executive control? Trends in Cognitive Sciences, 13, 160–166. 10.1016/j.tics.2009.01.006 19285913 PMC2773442

[c56] Phelps E. A. (2004). Human emotion and memory: Interactions of the amygdala and hippocampal complex. Current Opinion in Neurobiology, 14, 198–202. 10.1016/j.conb.2004.03.015 15082325

[c57] Radloff L. S. (1977). The CES-D scale a self-report depression scale for research in the general population. Applied Psychological Measurement, 1, 385–401. 10.1177/014662167700100306

[c101] Rogers G. M., Park J. H., Essex M. J., Klein M. H., Silva S. G., Hoyle R. H., . . . Pathak S. (2009). The dysfunctional attitudes scale: Psychometric properties in depressed adolescents. Journal of Clinical Child & Adolescent Psychology, 38, 781–789. 10.1080/15374410903259007 20183662

[c58] Rottenberg J. (2007). Major depressive disorder: Emerging evidence for emotion context insensitivity In Rottenberg J. & Johnson S. L. (Eds.), Emotion and psychopathology: Bridging affective and clinical science (pp. 151–165). Washington, DC: American Psychological Association 10.1037/11562-007

[c59] Schweizer S., & Dalgleish T. (2011). Emotional working memory capacity in posttraumatic stress disorder (PTSD). Behaviour Research and Therapy, 49, 498–504. 10.1016/j.brat.2011.05.007 21684525 PMC3145962

[c60] Schweizer S., & Dalgleish T. (2016). The impact of affective contexts on working memory capacity in healthy populations and in individuals with PTSD. Emotion, 16, 16–23. 10.1037/emo0000072 26414191 PMC7614023

[c61] Schweizer S., Satpute A. B., Atzil S., Field A. P., Barrett L. F., & Dalgleish T. (2016). The behavioral and neural effects of affective information on working memory performance: A pair of meta-analytic reviews. Manuscript submitted for publication. 10.1037/bul0000193 PMC6526745 31021136

[c62] Segal D. L., Coolidge F. L., Cahill B. S., & O’Riley A. A. (2008). Psychometric properties of the Beck Depression Inventory II (BDI-II) among community-dwelling older adults. Behavior Modification, 32, 3–20. 10.1177/0145445507303833 18096969

[c63] Segal Z. V., Kennedy S., Gemar M., Hood K., Pedersen R., & Buis T. (2006). Cognitive reactivity to sad mood provocation and the prediction of depressive relapse. Archives of General Psychiatry, 63, 749–755. 10.1001/archpsyc.63.7.749 16818864

[c64] Segal Z. V., Shaw B. F., Vella D. D., & Katz R. (1992). Cognitive and life stress predictors of relapse in remitted unipolar depressed patients: Test of the congruency hypothesis. Journal of Abnormal Psychology, 101, 26–36. 10.1037/0021-843X.101.1.26 1537969

[c65] Sheppard L. C., & Teasdale J. D. (2004). How does dysfunctional thinking decrease during recovery from major depression? Journal of Abnormal Psychology, 113, 64–71. 10.1037/0021-843X.113.1.64 14992658

[c66] Snyder H. R. (2013). Major depressive disorder is associated with broad impairments on neuropsychological measures of executive function: A meta-analysis and review. Psychological Bulletin, 139, 81–132. 10.1037/a0028727 22642228 PMC3436964

[c67] Tavitian L. R., Ladouceur C. D., Nahas Z., Khater B., Brent D. A., & Maalouf F. T. (2014). Neutral face distractors differentiate performance between depressed and healthy adolescents during an emotional working memory task. European Child & Adolescent Psychiatry, 23, 659–667. 10.1007/s00787-013-0492-9 24248754

[c68] Teasdale J. D. (1988). Cognitive vulnerability to persistent depression. Cognition and Emotion, 2, 247–274. 10.1080/02699938808410927

[c69] Vuilleumier P. (2005). How brains beware: Neural mechanisms of emotional attention. Trends in Cognitive Sciences, 9, 585–594. 10.1016/j.tics.2005.10.011 16289871

[c76] Wang C. E., Halvorsen M., Eisemann M., & Waterloo K. (2010). Stability of dysfunctional attitudes and early maladaptive schemas: A 9-year follow-up study of clinically depressed subjects. Journal of Behavior Therapy and Experimental Psychology, 41, 389–396. 10.1016/j.jbtep.2010.04.002 20452570

[c70] Weissman A. N., & Beck A. T. (1978 3 27–31). Development and validation of the Dysfunctional Attitude Scale: A preliminary investigation. Paper presented at the annual meeting of the American Educational Research Association, Toronto, Ontario, Canada Retrieved from http://files.eric.ed.gov/fulltext/ED167619.pdf

[c100] Wisco B. E. (2009). Depressive cognition: Self-reference and depth of processing. Clinical Psychology Review, 29, 382–392. 19346043 10.1016/j.cpr.2009.03.003

[c71] Yoon K. L., LeMoult J., & Joormann J. (2014). Updating emotional content in working memory: A depression-specific deficit? Journal of Behavior Therapy and Experimental Psychiatry, 45, 368–374. 10.1016/j.jbtep.2014.03.004 24747511

[c72] Zuroff D. C., Igreja I., & Mongrain M. (1990). Dysfunctional attitudes, dependency, and self-criticism as predictors of depressive mood states: A 12-month longitudinal study. Cognitive Therapy and Research, 14, 315–326. 10.1007/BF01183999

