# Supplementary material for: Affective Enhancement of Working Memory Is Maintained in Depression
Source: Emotion. 2017 Apr 13;18(1):127–37. doi: 10.1037/emo0000306 (PMC5819821; doi:10.1037/emo0000306)
Supplement: Supplementary file 1 [file Supplementary.docx]

**SM 1: Supplementary results and discussion for Experiment 2: Comparing individuals currently in a MDD episode to never-depressed controls**

Comparing performance of individuals currently suffering from MDD to never-depressed controls yielded no significant effect of valence, *F* (1, 27) = 2.85, *p* = .103, *η*_p_^2^ = .10, BF_01_ = 1.21, *p*(H1, *D*) = 0.45. Though the moderate effect size and Bayesian analyses suggest that this may be an artefact of the low power to detect an effect in this small sample, Power_Observed_ = .37. There was no effect of group on WMC *F* (1, 27) = 1.32, *p* = .261, *η*_p_^2^ = 0.05, BF_01_ = 1.73, *p*(H1, *D*) = 0.37.

**Table S1. WM performance across comparing individuals currently in a MDD episode to never-depressed controls**

|  | Never depressed  *n* = 14 | Current MDD  *n* = 13 |
| --- | --- | --- |
| Neutral *M* (*sd*) | .50 (.15) | .43 (.15) |
| Depressogenic *M* (*sd*) | .53 (.22) | .49 (.18) |

*Table S1* Neutral = proportion of words recalled correctly in the context of neutral sentences; Depressogenic = proportion of words recalled correctly in the context of depressogenic sentences; Never depressed = individuals with no history of MDD; Current MDD = individuals currently suffering from MDD.
